# Effects of a Multi-Ingredient Oral Supplement on Multiple Object Tracking, Reaction Time, and Reactive Agility

**DOI:** 10.1080/15502783.2022.2140014

**Published:** 2022-11-16

**Authors:** Justine M. Renziehausen, Amy M. Bergquist, Jeffrey R. Stout, Adam J. Wells, David H. Fukuda

**Affiliations:** a Physiology of Work & Exercise Response (POWER) Laboratory, Institute of Exercise Physiology and Rehabilitation Science, School of Kinesiology and Physical Therapy, College of Health Professions and Sciences, University of Central Florida, Orlando, FL, USA; b Exercise Physiology Intervention and Collaboration (EPIC) Laboratory, Institute of Exercise Physiology and Rehabilitation Science, School of Kinesiology and Physical Therapy, College of Health Professions and Sciences, University of Central Florida, Orlando, FL, USA

**Keywords:** multi-ingredient supplements, cognition, reaction time, reactive agility, caffeine

## Abstract

**Background:**

The demands of typical daily activities require a constant level of alertness and attention. Multi-ingredient, caffeine-containing supplements have been shown to improve measures of cognitive performance. As many of these supplements become readily available, efficacy of each should be evaluated. Therefore, the purpose of this study is to examine the effects of the 4D dietary supplement on cognition, reaction time, and reactive agility.

**Methods:**

Seventeen healthy males (n = 8) and females (n = 9) between the ages of 18–40 years old (22.8 ± 2.9 years; 167.3 ± 9.6 cm; 65.4 ± 10.9 kg) participated in this double-blind, randomized crossover study. Participants completed three baseline reaction time assessments on the Dynavision and one baseline multiple object tracking assessment on the Neurotracker. Participants then consumed the oral multi-ingredient supplement containing 150 mg of caffeine or non-caffeinated placebo, mixed with 24 ounces of water, and rested for 45 minutes. Following the rest period, participants completed an additional three reaction time assessments and one multiple object tracking (MOT) assessment, as well as 6–12 trials of the Y-reactive agility test. Repeated measures ANOVAs were used to evaluate YRA performance and change values for Dynavision RT, Dynavision score, and MOT speed with either 4D dietary supplement or placebo.

**Results:**

A significant time × supplement interaction was shown for MOT speed (p = .040, *d = *.543). Change scores in MOT speed were significantly different from zero following 4D (mean: 0.224 au; 95% confidence interval: 0.050 to 0.398 au) but not placebo supplementation (mean: −0.046 au; 95% confidence interval: −0.220 to 0.127 au). No time × supplement interaction was shown for Dynavision RT (p = .056, *d* = −.499) or Dynavision score (p = .093, *d* = .434). No differences were shown for YRA scores following supplementation for the right side (p = .241, *d = −.2*95) or left side (p = .378, *d = −.2*20).

**Conclusion:**

The 4D dietary supplement appears to improve measures of cognition, specifically attention/spatial awareness, but not reaction time or reactive agility. Future research should examine the effects of this supplement with a larger, less heterogeneous sample and/or in conjunction with an exercise intervention.

## 1. Introduction

The everyday demands of work, family, and activities of daily living require complex decision-making, a constant level of alertness, and sustained attention [1]. Using nutritional supplements to ensure optimal cognitive performance throughout daily tasks is becoming far more common, especially as the supplement industry continues to grow [2,3]. More recently, pre-workout and other multi-ingredient supplements are gaining popularity as they contain several ingredients that claim to provide synergistic benefits on cognitive performance [2]. As more of these products continue to become available, it may be difficult to determine which have proven efficacy for the intended purpose.

The term ‘cognition’ encompasses many domains, including attention, reaction time, working memory, vigilance, alertness, judgment, and decision making [1]. Spatial awareness potentially represents a subset of these abilities applied when an individual interacts with the surrounding environment [4,5]. The Neurotracker is a device designed to engage spatial awareness through specific aspects of attention (selective, distributed, dynamic, and sustained) and three-dimensional multiple object tracking (MOT) during a perceptual-cognitive task [6]. Previous literature suggests that multiple object tracking may apply to daily functions such as driving or improving working memory [6,7]. While previous literature has shown positive effects of multi-ingredient supplements on simple cognitive assessments (such as reaction time) in static situations, few studies have examined the effects of supplementation on dynamic cognitive assessments, such as multiple object tracking or reactive agility tests, that allow for quantitative assessments of spatial awareness and decision-making skills while in motion.

One of the most common and well-studied components in multi-ingredient supplements is the central nervous system stimulant caffeine [3,8]. Caffeine is widely known to improve various components of athletic performance including aerobic, sprint, and resistance training performance [8,9] but has also been shown to improve alertness, as well as several aspects of cognition [8–10], including reaction time [8,11,12], attention/vigilance [13], and memory [10]. Although several studies report positive effects of caffeine on cognition, others report no change or even negative results due to side effects such as anxiety, restlessness, acute increases in blood pressure, and feelings of jitteriness [1,14,15]. In theory, multi-ingredient supplements are designed to enhance performance while mitigating potential unwanted adverse effects synergistically [2,11,12,15].

For example, it has been reported that ingesting 150 mg of caffeine, in combination with 250 mg L-theanine, can significantly improve reaction time more than 150 mg of caffeine alone or the placebo [14]. Additionally, acute supplementation with Ginseng, a common psychoactive herbal supplement, has been shown to positively affect various measures of cognition [16]. It has also been shown that tyrosine is an effective ingredient for improving measures of cognition, mood, and attention [17]. Few studies have examined the effects of tyrosine and caffeine together, although there is potential for positive combined effects [17]. Improving cognition and reaction time and overall physical performance measures may be beneficial for a variety of populations.

Depending on dosage, caffeine consumption alone may lack the ability to enhance cognition and mood [2,11], and therefore, multi-ingredient supplement formulations could offer alternative options to enhance performance [2,11]. One study examined the effects of a multi-ingredient supplement containing caffeine, L-theanine, L-citrulline, and ashwagandha and found it effective at increasing cerebral-cortical activation (a measure of cognition) and reaction time [2]. Further, a review of the potential ergogenic benefits of multi-ingredient supplements indicated positive effects on subjective measures of fatigue and alertness [12]. Although implications for using multi-ingredient supplements are generally positive, the methodologies and combination of ingredients vary extensively. Recently, an oral multi-ingredient supplement (4D dietary supplement) was created with the intent to benefit all populations by providing nutritional value (vitamins, electrolytes) and improving cognition, reaction time, and alertness. Therefore, the purpose of this study was to examine the effects of the 4D dietary supplement on a series of cognitive assessments and reaction times.

## 2. Methods

### Study Design

This study followed a double-blind, randomized crossover design. Participants were asked to report to the laboratory on five separate occasions. The first visit consisted of informed consent and screening questionnaires (Physical Activity Readiness Questionnaire (PAR-Q+) and the medical health history questionnaire (MHQ), and the caffeine consumption questionnaire (CCQ)) to determine eligibility. The next two visits were familiarization days. On Visit 1, participants were asked to complete a demographics questionnaire, followed by height, weight, and body composition assessments. Participants were also asked to complete eight familiarization trials of the Mode A reaction time (RT) assessment on the Dynavision D2 and 2–3 trials of the Y-Reactive Agility test (YRA). On Visit 2, participants were asked to complete an additional 8 familiarization trials on the Dynavision D2 Mode A assessment, three core sessions of the Neurotracker multiple object tracking (MOT) assessment, and three additional trials of the YRA. On Visits 3 and 4, participants completed baseline RT (3 trials) and MOT (one core session) assessments. Upon completing these assessments, participants were given either the 4D dietary supplement or placebo and instructed to mix the powder with 24 ounces of water. After participants finished the drink, they were instructed to rest for 45 minutes. Following the rest period, participants were asked to complete additional RT (3 trials) and MOT (one core session) assessments. Participants then completed a standardized warm-up, followed by approximately 6–15 trials of the YRA. After receiving an explanation of all procedures involved in this study, informed consent was obtained. All procedures were approved by the University of Central Florida’s Institutional Review Board (00003029). This study was registered with clinicaltrials.gov under the identifier NCT04988919.

### Participants

A convenience sample of healthy individuals was recruited from the University of Central Florida to participate in this study. Participants were required to be healthy as determined by the PAR-Q+ and the MHQ to have a daily caffeine consumption less than or equal to 200 mg, as determined by the Caffeine Consumption Questionnaire [18]. Participants were instructed to maintain the same sleep, physical activity, and caffeine consumption habits between testing days. Twenty male (n = 10) and female (n = 10) participants completed the testing procedures; however, three individuals were excluded due to extended time between testing sessions (≥14 days). Therefore, the final analysis included 17 males (n = 8) and females (n = 9) between the ages of 18–40 years old (22.8 ± 2.9 years; 167.3 ± 9.6 cm; 65.4 ± 10.9 kg; 22.5 ± 5.8 BF%). These participants completed testing sessions within 7 days (4.4 ± 2.0 days).

## 3. Measures

### Anthropometrics

Height and weight measurements were assessed using a scale and stadiometer (Patient Weighing Scale, Model 500 KL, Pelstar, Alsip, IL). Body composition was assessed using a bioelectrical impedance spectroscopy analyzer (Sozo; Carlsbad, CA, USA). Prior to testing, participants were asked to come to the laboratory well-hydrated and fasted for at least 2 hours. Participants were asked to remove shoes, socks, and jewelry prior to anthropometric assessments. Participants stood on the scale for approximately 1 min.

### Multiple Object Tracking (MOT)

Visual tracking speed was assessed via MOT on the Neurotracker software (CogniSens Athletic Inc., Montreal, Canada). The software is designed to engage specific aspects of attention (selective, distributed, dynamic, and sustained) and motion tracking [6]. The MOT assessment requires participants to track moving objects on a 3D screen while wearing 3D glasses. Each core session, including familiarization, consisted of 20 trials. At the beginning of the test, eight balls appeared on the screen. Four balls randomly appeared white for 2 s and then returned to yellow. Participants were asked to visually track these four balls as all eight moved around the screen for 8 seconds. Following the 8 seconds, all balls froze on the screen and appeared with numbers (1–8). Participants verbally communicated to researchers the numbers of the balls that they were tracking. The speed of the balls on the first trial on testing days was standardized at 1.0, which corresponds to a speed of 68 cm/s. The speed of the balls in subsequent trials was contingent on correct identification of all four target balls, and adjusted in a staircase fashion (one up one down), as previously described [19]. MOT speed was recorded at the end of each session (20 trials). Participants completed this assessment a total of 7 times: three times on V2 for familiarization to establish a baseline (as recommended by NeurotrackerX), and twice on each testing day (V3 and V4), before and after supplementation. Test–retest reliability for MOT speed determined between trials 3 and 4 during the current investigation demonstrated an ICC_3,1_ value of 0.808.

### Reaction Time

The Dynavision D2 (Dynavision International LLC, West Chester, OH) was used to assess reaction time. The Dynavision is a 4 × 4 ft. board with 64 buttons arranged in 5 circles, with a small screen in the center. Each button can light up, providing a stimulus that participants must react to. Participants in this study completed Mode A, which begins with a 5-s countdown on the screen. Once the test began, a random button became illuminated until the participant struck it with their hand, at which time, another button on the board became illuminated randomly. Participants were instructed to strike as many buttons as possible in 60 seconds. Reaction time and number of hits were recorded. Participants completed this assessment a total of 28 times throughout the study: 16 familiarization trials (8 on V1 and 8 on V2), and 12 testing trials (3 times before and 3 times after consumption of the 4D dietary supplement and placebo on V3 and V4). Reaction time (RT) and score (based on number of buttons hit) were recorded. The number of familiarization trials is based on a recent study about establishing a true baseline on the Dynavision D2 device [20]. This study reported significant differences in results during the first 15 trials and all further trials resulted in no significant difference, with an ICC_2,3_ values of 0.933 for number of hits and 0.939 for average RT [20]. Test–retest reliability for score (number of hits) and RT between V2 and V3 (pre-supplementation) in the current investigation demonstrated ICC_3,1_ values of .911 and .907, respectively.

### Y-Reactive Agility Test

**Figure 1.**
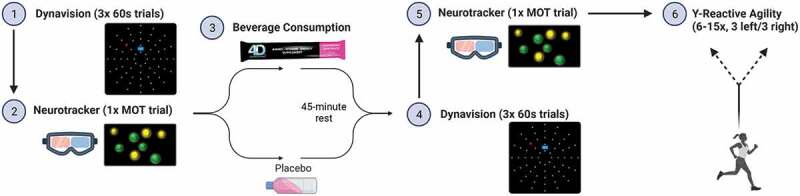
Event sequence on testing visits (V3 and V4). Created with BioRender.com.

The YRA is a sprint test that requires participants to change direction based on a light stimulus provided after the commencement of running. The Witty wireless timing gate system (Microgate USA, Mahopac, NY, USA) was used to record times and provide the change-of-direction stimulus to participants. Prior to beginning the test, participants performed a warm-up consisting of 5 minutes of jogging, lower body dynamic stretching, and four 10-m progressive runs (at 50%, 60%, 70%, and 90% perceived maximum effort). Participants began approximately 0.3 m behind the start line and were instructed to begin sprinting as fast as possible through the starting line, at which point time began to be recorded. Participants continued to sprint 5 m until the path split into two different directions. The timing gate system displayed an arrow pointing either left or right, and participants were asked to change direction based on this cue and finish sprinting 5 m to the finish line. The timing gate stopped recording time once participants crossed the finish line. A rest period of 2 minutes was provided between trials. Participants performed the 3 trials of the YRA on each familiarization day and 6–15 (as many as it took to get 3 arrows to each side) trials following supplementation on V3 and V4. Average values of the three trials on each side were used for statistical analyses. This assessment has been previously determined to be reliable, with a CV of 3% [21]. Test–retest reliability of YRA time between V1 and V2 in the current investigation demonstrated an ICC_3,1_ value of .853. The sequence of events on testing visits (V3 and V4) is displayed in Figure 1.

### Supplementation

**Figure 2.**
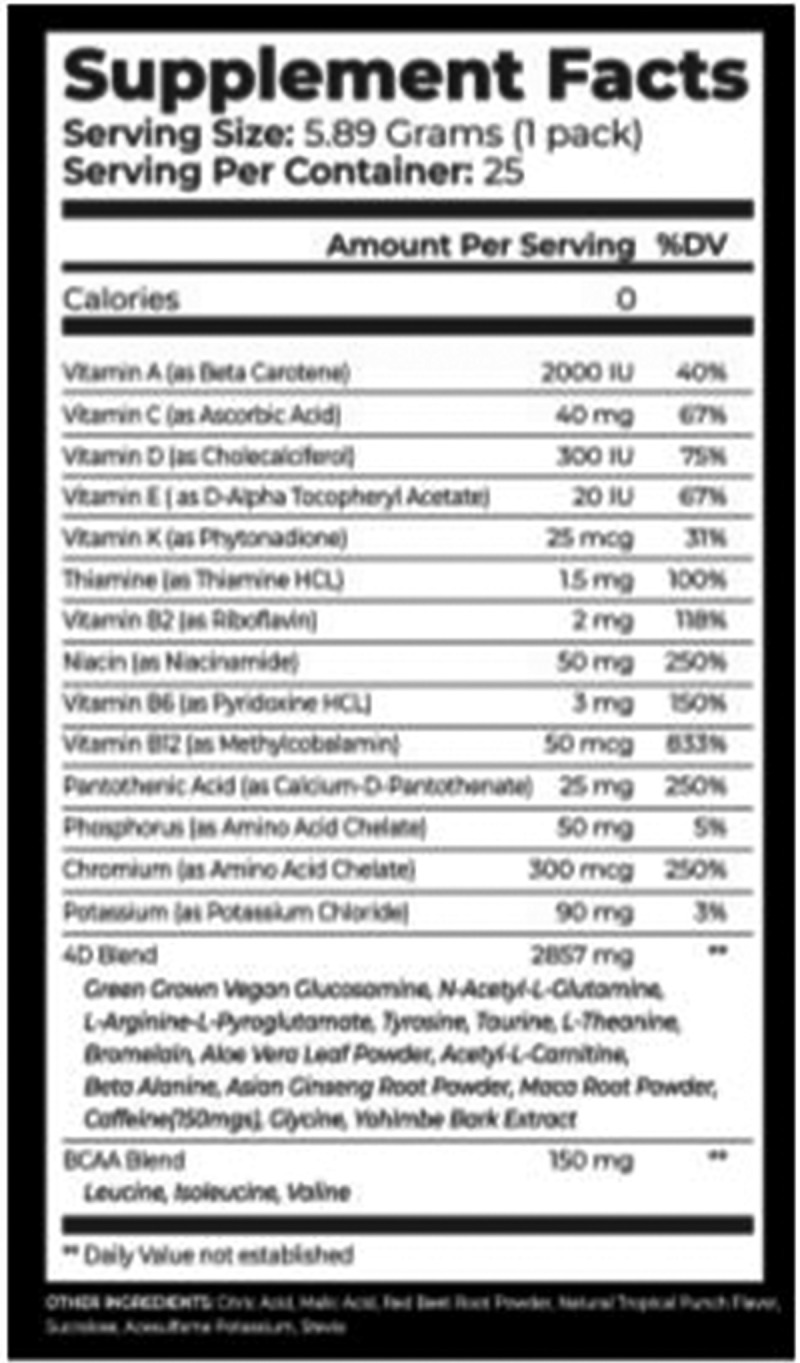
Nutritional information panel for the 4D dietary supplement.

Participants were asked to orally consume one pack (5.89 g) of the 4D dietary supplement (containing 150 mg caffeine) or a taste-matched placebo (raspberry lemonade Crystal Light®) mixed with 24 fluid ounces of water. Each participant was given a plastic, colored water bottle to help conceal any minor differences in drink appearances. The 4D dietary supplement list of ingredients and nutritional information is provided in Figure 2.

### Statistical analysis

Shapiro Wilk tests were used to assess normality. Paired sample t-tests were used to assess differences in sleep duration and caffeine consumption between testing days. Repeated measures ANOVAs were used to evaluate YRA performance and change values for Dynavision RT, Dynavision score, and MOT speed with either 4D or placebo. Additionally, repeated measures ANCOVAs were used to assess differences in all variables when accounting for centered body mass values. Cohen’s *d* effect sizes were used for further evaluation and interpreted as large (*d* > .80), moderate (*d* > .50), or small (*d* > .20) *[22]*. All statistical procedures were conducted using open-source statistical software (JASP 14.0.0) with a significance level set at p < .05.

## 4. Results

**Figure 3.**
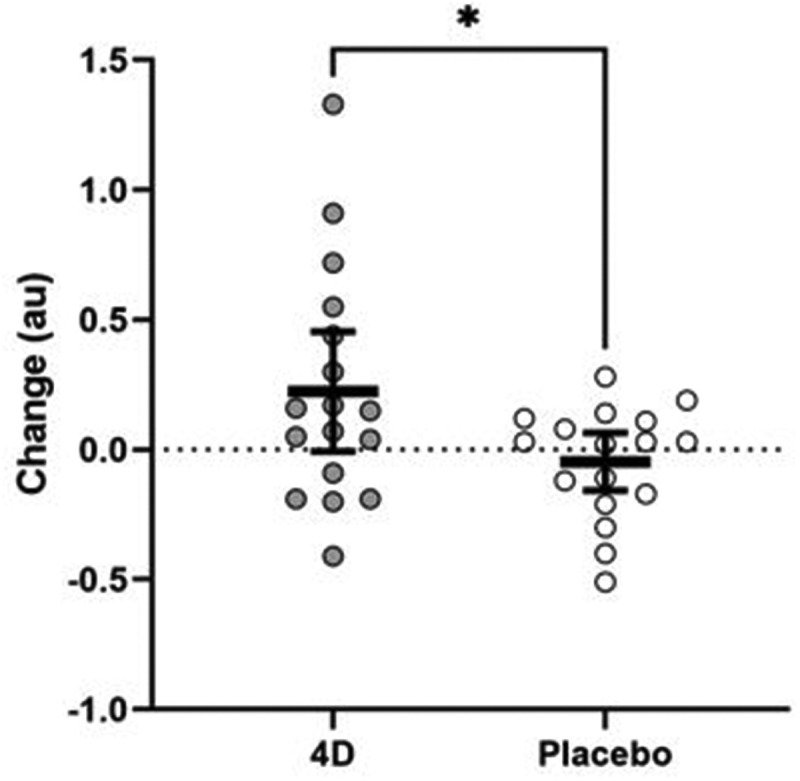
Changes in multiple object tracking speed following 4D and placebo supplementation. the thick black line depicts the mean, and the 95% confidence intervals is indicated by the end of the vertical error bar.

All dependent variables were normally distributed and no significant differences in sleep duration (t_16_ = 0.833, p = 0.417) or caffeine consumption (t_16_ = −1.000, p = 0.332) were found between testing visits. Table 1 provides the mean and standard deviation values for all dependent variables. A significant time × supplement interaction was shown for MOT speed with (F_1, 15_ = 4.76, p = 0.045, *d = *0.527) and without accounting for body mass (F_1,16_ = 5.02, p = 0.040, *d = 0*.543) (Figure 3). Change scores in MOT speed were significantly different from zero following 4D (mean: 0.224 au; 95% confidence interval: 0.050 to 0.398 au) but not placebo supplementation (mean: −0.046 au; 95% confidence interval: −0.220 to 0.127 au).

**Table 1.** Pre- and post-supplementation values.

		4D			Placebo		
		Mean		SD	Mean		SD
Multiple object tracking speed (au)*	Pre	1.26	±	0.36	1.41	±	0.51
	Post	1.48	±	0.46	1.36	±	0.44
Dynavision reaction time (s)	Pre	0.641	±	0.052	0.636	±	0.046
	Post	0.635	±	0.047	0.644	±	0.049
Dynavision score (# of touches)	Pre	94.3	±	7.8	94.8	±	7.3
	Post	94.9	±	7.3	93.9	±	7.6
Y-reactive agility (s)	Left	2.96	±	0.18	3.00	±	0.23
(Post-only values)	Right	2.96	±	0.17	2.98	±	0.22

*Indicates that change scores between 4D and placebo are significantly different (p < .05)

**Figure 4.**
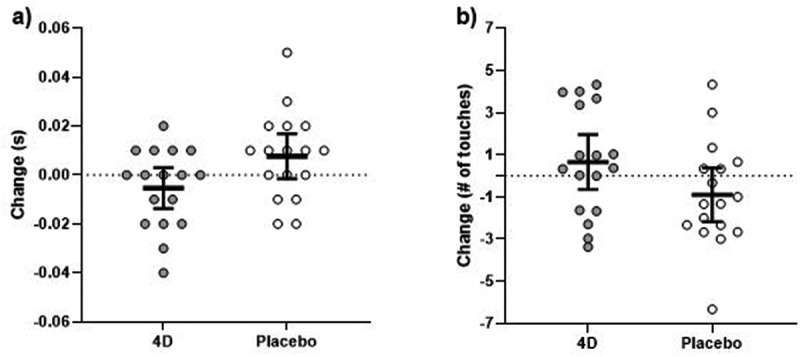
Changes in a) Dynavision reaction time and b) Dynavision score following 4D and placebo supplementation. the thick black line depicts the mean, and the 95% confidence interval is indicated by the ends of the vertical error bar.

No time × supplement interaction was shown for Dynavision RT with (F_1, 15_ = 3.96, p = 0.065, d = −0.568) and without accounting for body mass (F_1, 16_ = 4.24, p = 0.056, d = −0.499) (Figure 4A). No time × supplement interaction was shown for average Dynavision score with (F_1, 15_ = 2.82, p = 0.114, d = 0.489) and without accounting for body mass (F (1,16) = 3.20, p = 0.093, d = 0.434) (Figure 4B).

**Figure 5.**
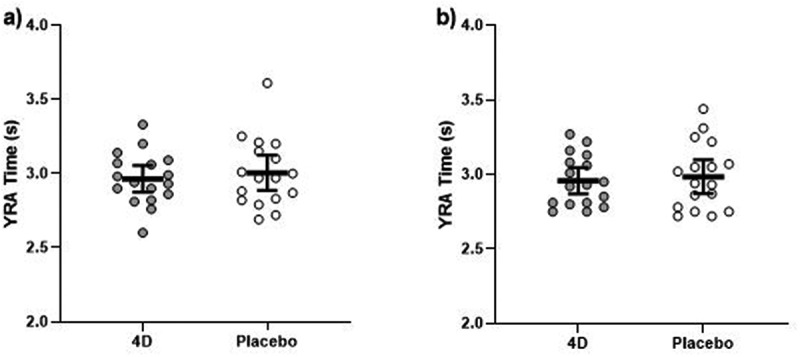
Y-reactive agility (YRA) time for the a) left and b) right sides following 4D and placebo supplementation. The thick black line depicts the mean, and the 95% confidence interval is indicated by the ends of the vertical error bar.

No differences were shown for YRA scores following supplementation for the right side (F_1, 16_ = 1.48, p = 0.241, *d = −0*.295) or left side (F_1, 16_ = 0.822, p = 0.378, *d = −0*.220) (Figure 5). When accounting for body mass, no time × supplement interaction was shown for YRA scores following supplementation for the right side (F_1, 15_ = 1.09, p = 0.314, *d = −0*.336), or left side (F_1, 15_ = 1.24, p = 0.283, *d = −0*.226).

## 5. Discussion

The purpose of this study was to determine if the 4D dietary supplement influenced performance on the Neurotracker (to assess attention/spatial awareness via MOT/visual tracking speed), Dynavision (to assess reaction time), and the y-reactive agility test (to assess reactive agility). It was hypothesized that consuming the oral supplement would improve reaction time, spatial awareness/MOT, and reactive agility. Results of this study indicate that the 4D supplement significantly improved MOT/visual tracking speed. However, there was no significant difference in reaction time or reactive agility between supplement and placebo.

These findings are in line with previous literature that discusses the effects of caffeine and multi-ingredient supplements on measures of cognition, specifically attention tasks. One study considered the effects of an energy drink containing 80 mg caffeine, B-group vitamins, and 1000 mg taurine on measures of cognition, concluding that the energy drink significantly improved memory performance and the speed of information retrieval from two memory tasks [23]. Another study considered the effects of a multi-ingredient supplement containing β-alanine, 284 mg caffeine, N-acetyl-L-tyrosine, and vitamins (e.g. Vitamin B_12_, Vitamin C, niacin, etc.). The authors concluded that although leg press and bench press performance were unaffected, improvements in cognitive function (via the Stroop test) and perceptions about readiness to perform exercise were present [24].

In previous studies examining the effect of multi-ingredient supplements on cortisol, mood, and cognitive assessments, it was determined that caffeine was the driving factor influencing improvements on cognition assessments (e.g. attention and memory) [25]. While caffeine (in doses ranging from .5 to 4 mg/kg) is generally considered effective for a variety of cognitive performance assessments, such as attention, alertness, short-term memory, vigilance, and sustained concentration [1,8], multi-ingredient supplements may improve measures of cognition to a greater extent than caffeine alone [26]. Similar results were presented in another pilot study examining the effects of caffeine vs. a multi-ingredient supplement without caffeine [27], and it was concluded that both supplements significantly improved measures of cognition. However, it should be noted that this was a pilot study, and the authors indicated the need for a greater sample size [27].

While caffeine is known to have various beneficial effects, this depends on factors such as participant habituation and weight [8]. Typically, caffeine is recommended in moderate doses between 3 and 6 mg/kg of body weight for performance improvements [8,9]. However, when consuming multi-ingredient supplements, participants typically receive one standardized dose. In the current study, participants received 150 mg of caffeine, which equated to <3 mg/kg body weight on average for all participants. This low dosage and varying levels of caffeine habituation [1,8,9] may have been partially responsible for the lack of significance in reaction time and YRA assessments. Additionally, individuals may metabolize caffeine differently based on genetic variations (e.g. CYP1A2 and ADORA2A), which may impact the effectiveness of caffeine supplementation [28].

It should be noted, however, that although results were not significant, there was a trend present for Dynavision reaction time measures (p = .056) with a moderate effect size, indicating the potential for improved reaction time following supplementation with the 4D dietary supplement. Post hoc power analysis for Dynavision reaction time using power analysis software (G*Power 3.1.9.4, HHU, Dusseldorf, Germany) revealed that for a within subjects repeated measures ANOVA, power of 0.80, p-value of 0.05, and an effect size (f) of 0.514 derived from the present study, the minimum necessary sample size is approximately 20. This is in line with previous research that shows positive effects of lower dose caffeine supplementation (3 mg/kg) on reaction time [29]. Additionally, it has been shown that both caffeine and a multi-ingredient supplement positively influenced YRA performance. However, both supplements contained a moderate dose (5 mg/kg) of caffeine [30]. A higher dose of caffeine compared to the standard amount in the 4D dietary supplement may be necessary to elicit significant changes in YRA performance.

A few limitations to the current investigation should be discussed. First, it is difficult to determine the specific dosage and ingredients that may be contributing to improved cognition and potentially improved reaction time assessments. While many of the ingredients (e.g. beta-alanine, caffeine, tyrosine, taurine, branched-chain amino acids, and yohimbe) found in this supplement are also prevalent in the top 100 most common multi-ingredient supplements [3], they may not be at the dosages necessary to elicit desired effects for all populations. The 4D dietary supplement is a proprietary blend, which limits the ability to compare dosages in this supplement to known efficacious doses. Furthermore, the active ingredient amounts in the product used for this study were not independently verified by a 3^rd^ party laboratory. Additionally, this study featured a heterogeneous sample including men and women, with varying training statuses, caffeine consumption habits, and experience with multi-ingredient supplements that could have influenced the results.

In conclusion, the 4D dietary supplement appears to improve measures of cognition (specifically aspects of attention/spatial awareness), as assessed by visual tracking speed. It may also aid in improving reaction time; however, more research is needed for conclusive results. These results have implications for a variety of populations, as most individuals can benefit from improved cognitive performance. For example, spatial awareness may assist athletes in tracking opponents, teammates, and a ball at once during competitions [19,31]. Additionally, as the aging process occurs, perceptual-cognitive abilities tend to decline [5], and various interventions can be considered to attempt to attenuate age-related cognitive deficits. For example, it has been previously demonstrated that a 6 week, full-body resistance training program has the ability to improve spatial awareness in older adults [5]. Future research may consider implementing a resistance training protocol in combination with a multi-ingredient supplement for optimal results. The effects of the 4D dietary supplement on reaction time and other aspects of cognition in more specific (e.g. athletes and older adults) and larger populations could also be explored.
